# Medical News

**Published:** 1854-07

**Authors:** 


### MEDICAL NEWS.

MEDICAL DEPARTMENT OF PENNSYLVANIA COLLEGE.—We learn, with much pleasure, that the Trustees of this Institution have lately appointed DRS. ALFRED STILLÉ and JOHN NEILL to the Chairs of *Practice of Medicine* and *Surgery*. These selections are eminently judicious, and must greatly strengthen the School. Dr. Stillé is one of the physicians of St. Joseph's Hospital, of this city, and was for many years

Lecturer on the Practice of Medicine in the Philadelphia Association for Medical Instruction, in both of which positions he has acquired a high reputation as a sound and eloquent teacher. Dr. Stillé is also well known to the profession as a frequent contributor to our medical literature. His work on Pathology is highly esteemed, and his Report on Medical Literature, presented some years ago to the American Medical Association, will long be remembered as one of the most brilliant papers ever recorded in its Transactions.

Dr. NEILL, we think we may safely say, combines many qualifications for the responsible chair which he has accepted. He was for several years Demonstrator of Anatomy in the University of Pennsylvania, and there established a reputation as one of the best anatomists and lecturers in our country. For some years he has occupied the situation of Surgeon to the Pennsylvania Hospital, and has delivered several courses on Clinical Surgery. From his position in this great school of Surgery, and from his long experience as a lecturer both on Anatomy and Surgery, Dr. NEILL's success as a Lecturer on the Principles and Practice of Surgery may be confidently predicted.

The late partial re-organization of the Medical Department of Pennsylvania College appears to us to have resulted in the construction of a Faculty of great strength. Prof. GILBERT has been at his own request, we learn, transferred from the Chair of Surgery to that of Obstetrics, in which department his large practical experience must give him great weight as a teacher. The branches of Anatomy, Chemistry, Institutes, and Materia Medica, in the hands of Profs. ALLEN, REESE, F. G. SMITH, and BIDDLE, will not suffer by comparison with any other institution. And we are well assured that the energy, ability, and industry, which are now centred in this organization, must soon place it in the foremost rank among the medical schools.

---

MEDICAL COLLEGE OF VIRGINIA.—The visiters appointed by the State Legislature for the governance of this Institution, have selected Dr. BEVERLY R. WELLFORD, of Fredericksburg, to fill the Chair of Materia Medica, and M. BROWN SÉQUARD as Professor of the Institutes of Medicine. Both of these gentlemen are favorably known to the profession throughout the United States, Dr. Wellford as President of the American Medical Association during the session of 1852, and M. Séquard as lecturer on Physiology, to which department of science he has added many original and valuable contributions.

Dr. G. A. Otis having removed from the State has resigned the editorship of the Virginia Medical and Surgical Journal. The readers of the Journal have cause to regret the retirement of so able and sprightly a writer. Dr. J. F. Peebles, of Petersburg, associated with Dr. J. B. McCaw, are the present editors. We most heartily wish them success in their undertaking.

—

The following extract from the editorial of Dr. H. D. Bulkley, of the New York Medical Times on the late meeting of the American Medical Association, evinces so kind and courteous a spirit towards our city, that we take great pleasure in laying it before our readers :—

"The only occurence which seems to have at all disturbed the harmony of the meeting was the transfer of the Publication Committee from Philadelphia to this city ; the discussion of which is said to have elicited some personal and sectional feeling, while the decision led to the offer of the resignation, by his colleagues, of Dr. Condie, as Treasurer of the Association. We regret this measure on many accounts, the more especially as it will have a tendency to mar the cordial feeling which should exist between the sister cities, and will be particularly unpleasant when we visit them, as many of us hope to do, next year, and partake of their hospitalities. The Association at first refused to accept the report of the Nominating Committee in favor of the change ; but it was subsequently adopted, after a second long and rather warm discussion in the committee of the whole. The change is said to have been made in accordance with the expressed wishes of the New York Delegation ; and if so, we cannot believe that they fairly represented the wishes of their constituents. At least, we have never heard of any expression of opinion to that effect, and know that the measure is regretted by some of the strong friends of the Association among us.''

———

In the "Bulletin Bibliographie," No. 4, of April 1854, an advertising sheet of the new books published in Paris, we find (page 5, No. 393,) the following."

Mémoire sur le traitment des fractures non réunies et des difformités des os, par Daniel Brainard. 1 vol. in 8, 3 fr.

In the "Feuilleton du Journal de la Librairie," No. 18, of May 6th, 1854, the same treatise is thus advertised—" Memoire sur le traitment des Fractures non réunies et des difformités des os, par le docteur D. Brainard, professeur de Chirurgie au Collége Médical de l'Illinois, à Chicago, Chirurgien de l'hôpital général, etc. Grand in 8v. de 72 pages, avec 2 planches gravées . . . 3.00. Is this the same treatise to which the American Medical Association awarded a prize at its last session, entitled "an Essay on a new method of treating Ununited fractures and certain Deformities of the Osseous system ?"

